# Supplementary material for: Gabija restricts phages that antagonize a conserved host DNA repair complex
Source: bioRxiv. 2025 Aug 30:2025.08.30.673261. Preprint. [Version 1] doi: 10.1101/2025.08.30.673261 (PMC12407980; doi:10.1101/2025.08.30.673261)
Supplement: Supplement 1 [file media-1.pdf]

Supplemental Data

Figure S1 Gabija significantly improves in phage restriction at 30ºC compared to 37ºC.

Figure S2 Complementation of Gabija's escape phage targeting with *orf53-65*. A $Gabija_{Hi}$ strain containing *orf66* plasmid is not viable.

Figure S3 Complementation of Gabija's escape phage targeting with Erf or Exo-Erf.

Figure S4 Gabija does not restrict escape phages in PAO1::*recA*$^{-}$.

Table S1 Predicted domains or homologs of D3 *orfs* in escaper deletions.

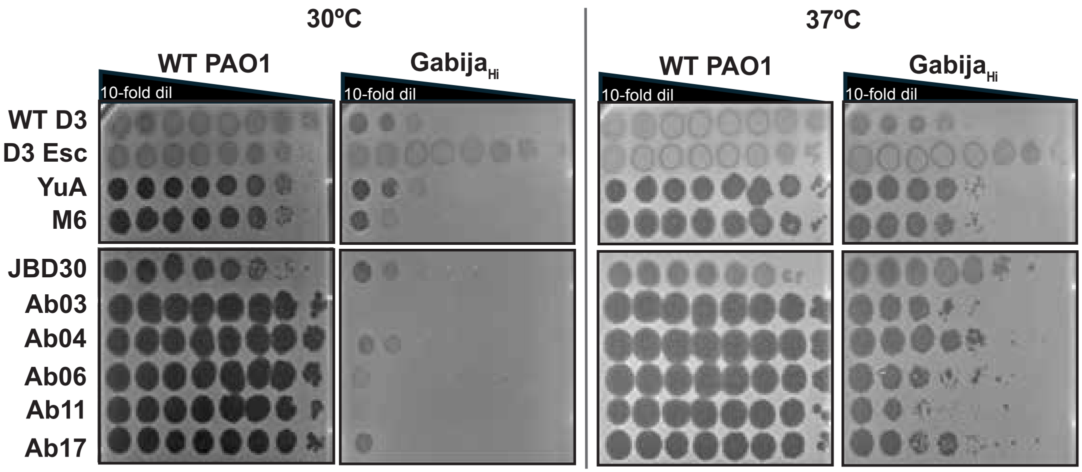
30ºC
37ºC
WT PAO1
Gabija$_{Hi}$
WT PAO1
Gabija$_{Hi}$
10-fold dil
10-fold dil
10-fold dil
10-fold dil
WT D3
D3 Esc
YuA
M6
JBD30
Ab03
Ab04
Ab06
Ab11
Ab17

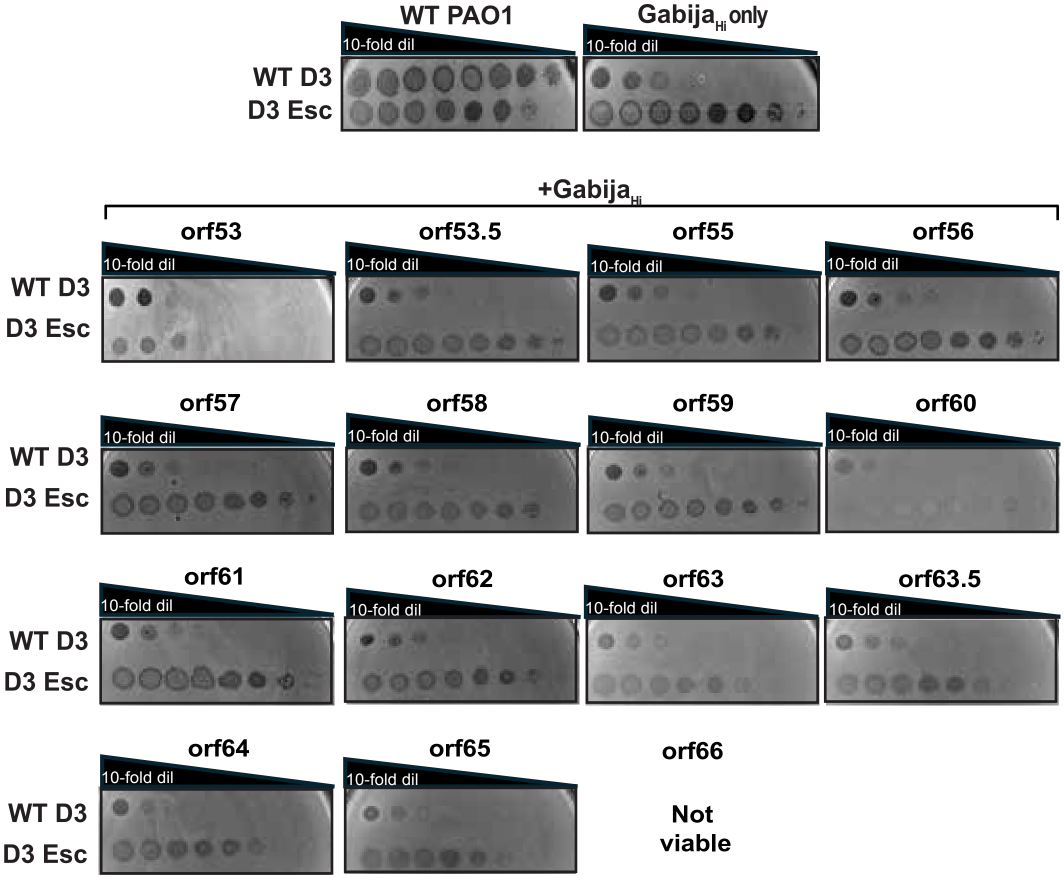
WT PAO1
Gabija$_{Hi}$ only
10-fold dil
WT D3
D3 Esc
+Gabija$_{Hi}$
orf53
orf53.5
orf55
orf56
orf57
orf58
orf59
orf60
orf61
orf62
orf63
orf63.5
orf64
orf65
orf66
Not viable

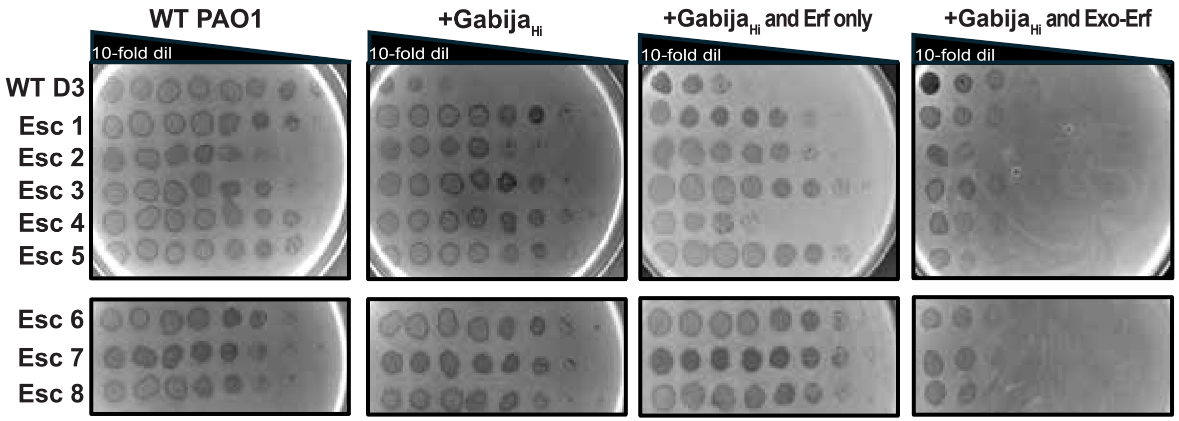
WT PAO1
+Gabija$_{Hi}$
+Gabija$_{Hi}$ and Erf only
+Gabija$_{Hi}$ and Exo-Erf
10-fold dil
WT D3
Esc 1
Esc 2
Esc 3
Esc 4
Esc 5
Esc 6
Esc 7
Esc 8

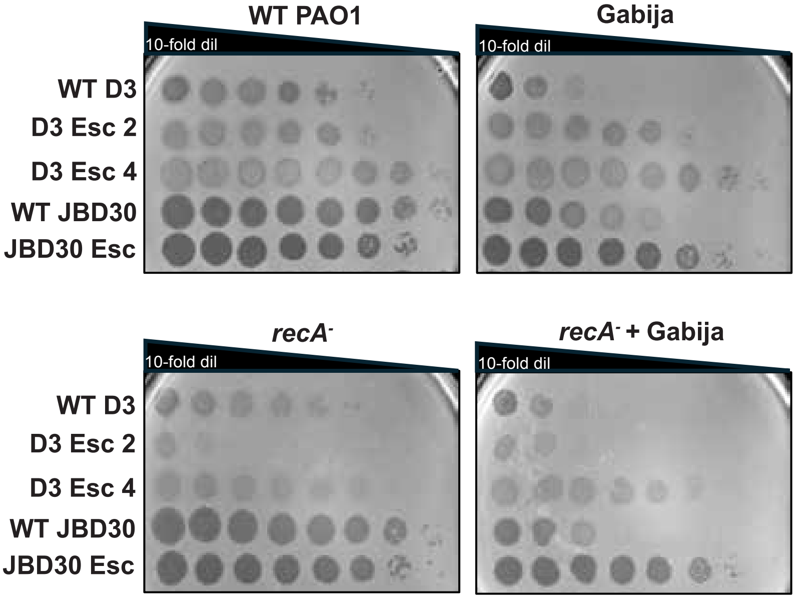
WT PAO1
Gabija
recA-
recA- + Gabija
10-fold dil
WT D3
D3 Esc 2
D3 Esc 4
WT JBD30
JBD30 Esc
